# Crystal structure of a new polymorph of di(thio­phen-3-yl) ketone

**DOI:** 10.1107/S2056989017013342

**Published:** 2017-09-29

**Authors:** Jörg Hübscher, André U. Augustin, Wilhelm Seichter, Edwin Weber

**Affiliations:** a TU Bergakademie Freiberg, Leipziger Strasse 29, D-09596 Freiberg/Sachsen, Germany

**Keywords:** crystal structure, di(thio­phen-3-yl) ketone, polymorphism, C—H⋯O hydrogen bonding, C—H⋯π inter­action

## Abstract

The crystal structure of the title compound, C_9_H_6_OS_2_, represents a new polymorph. The crystal structure was solved in the ortho­rhom­bic space group *Pbcn* with one half of the mol­ecule in the asymmetric unit. The thio­phene rings are perfectly planar and twisted with respect to each other, showing the mol­ecule to be in an S,O-*trans*/S,O-*trans* conformation. In the crystal, C—H⋯O hydrogen bonds connect the mol­ecules into layers extending parallel to the *ab* plane. The crystal structure also features π–π inter­actions.

## Abstract 2

A new polymorph of di(thio­phen-3-yl) ketone differing from the previous structure by the mol­ecular assembly is reported and comparatively discussed.

## Chemical context   

With reference to the principle of bioisosterism (Lima & Barreiro, 2005 ▸), thio­phene is an important structural moiety replacing benzene rings in drugs and biomolecules. Moreover, thio­phene is a highly polarizable group due to the presence of the π-electrons and the sulfur atom available in the ring, making it a structural unit worthy of investigation related to crystal engineering (Desiraju *et al.*, 2012 ▸). This involves potential π-stacking (Tiekink & Zukerman-Schpector, 2012 ▸) and C—H⋯π (Nishio *et al.*, 2009 ▸) inter­actions, as well as other contacts including a chalcogen atom such as sulfur (Gleiter *et al.*, 2003 ▸). From this point of view, the title compound is likely to be an inter­esting study object. However, searching in the literature shows its crystal structure being already described twice (Sheldrick *et al.*, 1978 ▸; Benassi *et al.*, 1989 ▸). On the other hand, a polymorph resulted from our work, the structure of which is reported here and comparatively discussed in connection with the previous findings, bearing in mind the attention currently attracted by the field of polymorphism in mol­ecular crystals (Bernstein, 2002 ▸; Cabri *et al.*, 2007 ▸; Braga *et al.*, 2009 ▸).
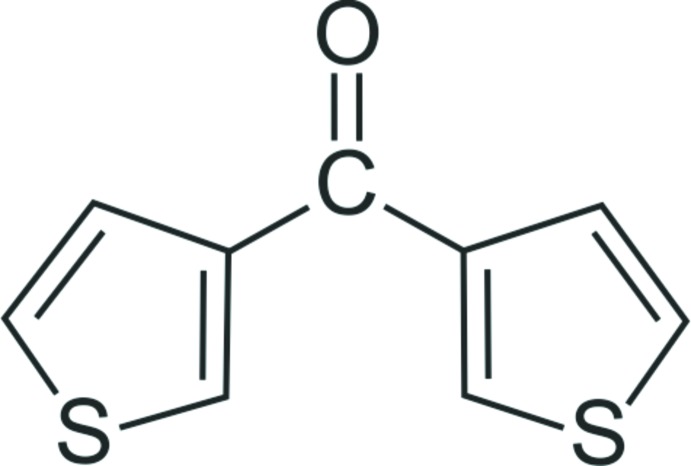


## Structural commentary   

**Figure 1:**
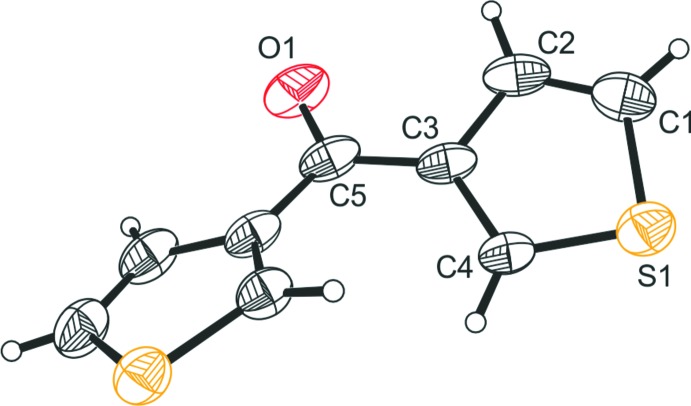
Perspective view of the mol­ecular structure of the title compound. Anisotropic displacement ellipsoids are drawn at the 30% probability level.

The title compound crystallizes in the space group *Pbcn* with one half of the mol­ecule in the asymmetric unit, *i.e.* the mol­ecule is located on the twofold symmetry axis. A perspective view of the mol­ecular structure of the title compound is presented in Fig. 1 ▸. The bond distances within the mol­ecule agree with those found in the reported crystal structures of the polymorphs of this compound (Sheldrick *et al.*, 1978 ▸; Benassi *et al.*, 1989 ▸). Taking into account experimental error, the thio­phene rings are perfectly planar. The heteroatom of the ring is always on the opposite side with respect to C=O, showing the mol­ecule to be in an S,O-*trans*/S,O-*trans* conformation, as was predicted to be the more stable conformation for the compound (Benassi *et al.*, 1989 ▸). The torsion angle along the atomic sequence O1—C5—C3—C4 is −155.2 (3)° and corresponds to an inter­planar angle of 42.3 (1)° between the thio­phene rings, being ascribed to steric hindrance between the H atoms on C4 and C4′.

## Supra­molecular features   

**Figure 2:**
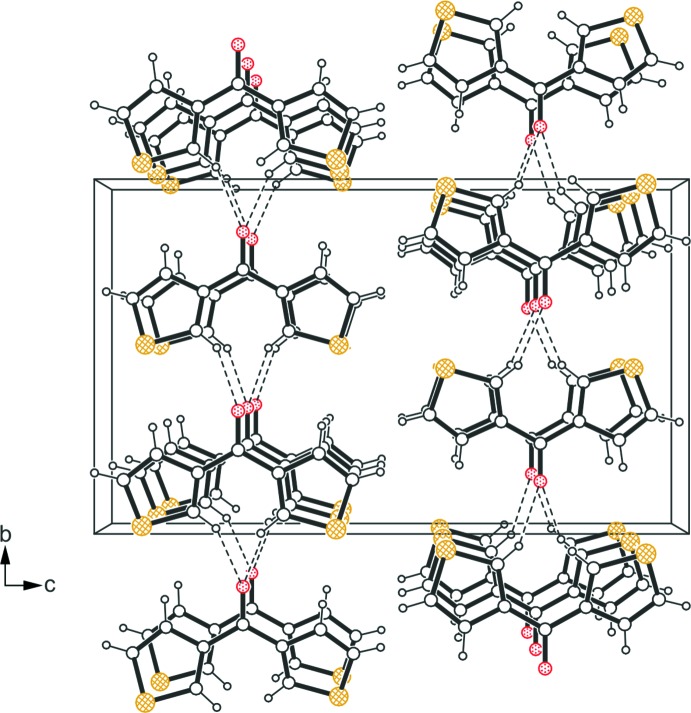
Packing diagram of the title compound viewed down the *a* axis. Dashed lines represent hydrogen-bonding inter­actions.

The crystal structure is composed of mol­ecular layers extending parallel to the *ab* plane (Table 1 ▸, Fig. 2 ▸). Within a given layer the mol­ecules are connected *via* C—H⋯O hydrogen bonds (Desiraju & Steiner, 1999 ▸) in which the oxygen atom acts as a bifurcated acceptor. Moreover, the layer structure features π–π stacking (Tiekink & Zukerman-Schpector, 2012 ▸) with a centroid⋯centroid distance of 3.946 (2) Å and a slippage of 1.473 Å between the inter­acting thio­phene rings. No directed non-covalent bonding is observed between the mol­ecules of consecutive layers, so that the crystal structure appears to be stabilized only by van der Waals forces in the stacking direction of the mol­ecular layers.

## Database survey   

**Figure 3:**
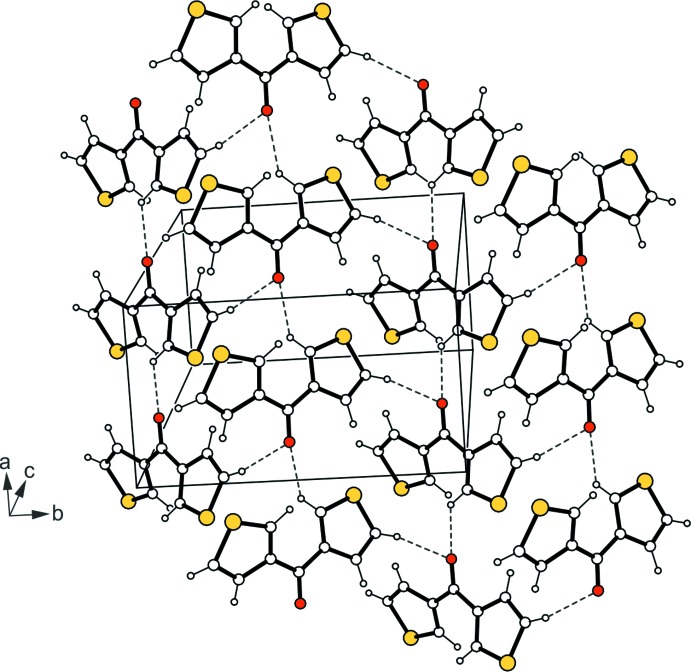
Packing excerpt of the previously reported polymorph (Benassi *et al.*, 1989 ▸). Hydrogen-bonding inter­actions are shown as dashed lines.

A search in the Cambridge Structural Database (CSD, Version 5.38, update February 2017; Groom *et al.* 2016 ▸) revealed two crystal structures of the title compound [Refcodes DTHKET (Sheldrick *et al.*, 1978 ▸) and DTHKET01 (Benassi *et al.*, 1989 ▸)]. In these polymorphs (space group: *P*2_1_/*c*, *P*2_1_/*n*, *Z* = 4) the mol­ecules show slight conformational differences and one of their thio­phene rings is disordered over two positions. It is obvious that crystallization from different solvents may have a fundamental influence on the mol­ecular assembly in the solid-state structure, thus giving rise to polymorphism (Bernstein, 2002 ▸; Cabri *et al.*, 2007 ▸; Braga *et al.*, 2009 ▸). Unfortunately, the previous reports do not include information about the solvent used for crystallization of the compound and thus it is not possible to engage in a more qualified discussion of the facts. In the structures of the reported polymorphs, C—H⋯O hydrogen bonds connect the mol­ecules into undulating sheets, in which the oxygen atom acts as a bifurcated acceptor (Fig. 3 ▸). Inter­sheet association is accomplished by C—H⋯π contacts, resulting in a three-dimensional supra­molecular architecture. In summary, the structures of the two polymorphs differ basically in the mol­ecular assembly.

## Synthesis and crystallization   

The synthesis of the title compound has been reported by different groups and following different procedures (Gronowitz & Erickson, 1963 ▸; Pittman & Hanes, 1977 ▸; Lucas *et al.*, 2000 ▸). We used the method of Lucas *et al.* ▸, reacting thio­phen-3-yl lithium (prepared from 3-bromo­thio­phene and *n*-BuLi in dry diethyl ether/*n*-hexane at 195 K under argon) with *N,N*-di­methyl­carbamoyl chloride. Column chromatography on SiO_2_ with *n*-hexa­ne/ethyl acetate (10:1) followed by recrystallization from methanol yielded the title compound as colourless crystals, m.p. 353 K. Previous values for the m.p. are 345–346 K (Gronowitz & Erickson, 1963 ▸) and 346 K (Lucas *et al.*, 2000 ▸) pointing to polymorphic structures of the previously and presently isolated crystals.

## Refinement   

Crystal data, data collection and structure refinement details are summarized in Table 2 ▸. The hydrogen atoms were positioned geometrically and refined isotropically using the riding model with C—H = 0.93 Å and *U*_iso_(H) = 1.2*U*_eq_(C).

## Supplementary Material

Crystal structure: contains datablock(s) I. DOI: 10.1107/S2056989017013342/qm2119sup1.cif

Structure factors: contains datablock(s) I. DOI: 10.1107/S2056989017013342/qm2119Isup2.hkl

Supporting information file. DOI: 10.1107/S2056989017013342/qm2119Isup3.cml

CCDC reference: 1575296

Additional supporting information:  crystallographic information; 3D view; checkCIF report

## Figures and Tables

**Table 1:** Hydrogen-bond geometry (Å, °)

*D*—H⋯*A*	*D*—H	H⋯*A*	*D*⋯*A*	*D*—H⋯*A*
C4—H4⋯O1^i^	0.93	2.42	3.261 (4)	151

Symmetry code: (i) 
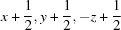
.

**Table 2:** Experimental details

Crystal data	
Chemical formula	C_9_H_6_OS_2_
*M*_r_	194.26
Crystal system, space group	Orthorhombic, *P* *b* *c* *n*
Temperature (K)	296
*a*, *b*, *c* (Å)	3.9464 (2), 11.5015 (5), 19.2970 (9)
*V* (Å^3^)	875.88 (7)
*Z*	4
Radiation type	Mo *K*α
μ (mm^−1^)	0.55
Crystal size (mm)	0.53 × 0.15 × 0.12

Data collection	
Diffractometer	Bruker APEXII CCD area detector
Absorption correction	Multi-scan (*SADABS*; Bruker, 2008 ▸)
*T*_min_, *T*_max_	0.759, 0.937
No. of measured, independent and observed [*I* > 2σ(*I*)] reflections	6469, 972, 667
*R*_int_	0.033
(sin θ/λ)_max_ (Å^−1^)	0.643

Refinement	
*R*[*F* ^2^ > 2σ(*F* ^2^)], *wR*(*F* ^2^), *S*	0.047, 0.161, 1.13
No. of reflections	972
No. of parameters	56
H-atom treatment	H-atom parameters constrained
Δρ_max_, Δρ_min_ (e Å^−3^)	0.35, −0.22

Computer programs: *APEX2* (and *SAINT* (Bruker, 2008 ▸), *SHELXS97* and *SHELXTL* (Sheldrick, 2008 ▸), *SHELXL2014* (Sheldrick, 2015 ▸) and *ORTEP-3 for Windows* (Farrugia, 2012 ▸).

## supplementary crystallographic information

### Crystal data

**Table:**

C_9_H_6_OS_2_	*D*_x_ = 1.473 Mg m^−^^3^
*M**_r_* = 194.26	Mo *K*α radiation, λ = 0.71073 Å
Orthorhombic, *P**b**c**n*	Cell parameters from 1847 reflections
*a* = 3.9464 (2) Å	θ = 3.2–24.5°
*b* = 11.5015 (5) Å	µ = 0.55 mm^−^^1^
*c* = 19.2970 (9) Å	*T* = 296 K
*V* = 875.88 (7) Å^3^	Plate, colourless
*Z* = 4	0.53 × 0.15 × 0.12 mm
*F*(000) = 400	

### Data collection

**Table:**

Bruker APEXII CCD area detector diffractometer	667 reflections with *I* > 2σ(*I*)
φ and ω scans	*R*_int_ = 0.033
Absorption correction: multi-scan (SADABS; Bruker, 2008)	θ_max_ = 27.2°, θ_min_ = 3.5°
*T*_min_ = 0.759, *T*_max_ = 0.937	*h* = −4→5
6469 measured reflections	*k* = −14→14
972 independent reflections	*l* = −24→24

### Refinement

**Table:**

Refinement on *F*^2^	0 restraints
Least-squares matrix: full	Hydrogen site location: inferred from neighbouring sites
*R*[*F*^2^ > 2σ(*F*^2^)] = 0.047	H-atom parameters constrained
*wR*(*F*^2^) = 0.161	*w* = 1/[σ^2^(*F*_o_^2^) + (0.0648*P*)^2^ + 0.649*P*] where *P* = (*F*_o_^2^ + 2*F*_c_^2^)/3
*S* = 1.13	(Δ/σ)_max_ < 0.001
972 reflections	Δρ_max_ = 0.35 e Å^−^^3^
56 parameters	Δρ_min_ = −0.22 e Å^−^^3^

### Special details

**Table:**

Geometry. All esds (except the esd in the dihedral angle between two l.s. planes) are estimated using the full covariance matrix. The cell esds are taken into account individually in the estimation of esds in distances, angles and torsion angles; correlations between esds in cell parameters are only used when they are defined by crystal symmetry. An approximate (isotropic) treatment of cell esds is used for estimating esds involving l.s. planes.

### Fractional atomic coordinates and isotropic or equivalent isotropic displacement parameters (Å^2^)

**Table:**

	*x*	*y*	*z*	*U*_iso_*/*U*_eq_	
S1	0.1430 (3)	0.46670 (8)	0.08955 (5)	0.0781 (4)	
O1	0.0000	0.1486 (3)	0.2500	0.1066 (15)	
C1	−0.0185 (11)	0.3364 (3)	0.0641 (2)	0.0791 (11)	
H1	−0.0650	0.3163	0.0184	0.095*	
C2	−0.0701 (9)	0.2660 (3)	0.1189 (2)	0.0755 (10)	
H2	−0.1562	0.1910	0.1148	0.091*	
C3	0.0202 (8)	0.3174 (2)	0.18397 (18)	0.0608 (8)	
C4	0.1397 (8)	0.4274 (2)	0.17415 (17)	0.0604 (8)	
H4	0.2116	0.4755	0.2100	0.073*	
C5	0.0000	0.2550 (3)	0.2500	0.0672 (13)	

### Atomic displacement parameters (Å^2^)

**Table:**

	*U*^11^	*U*^22^	*U*^33^	*U*^12^	*U*^13^	*U*^23^
S1	0.0824 (8)	0.0643 (6)	0.0875 (7)	−0.0016 (4)	0.0059 (5)	−0.0061 (4)
O1	0.136 (4)	0.0338 (15)	0.150 (4)	0.000	0.023 (3)	0.000
C1	0.077 (2)	0.072 (2)	0.088 (2)	0.005 (2)	−0.004 (2)	−0.029 (2)
C2	0.064 (2)	0.0496 (16)	0.113 (3)	−0.0041 (15)	−0.002 (2)	−0.0277 (18)
C3	0.0486 (17)	0.0383 (13)	0.096 (2)	0.0003 (12)	−0.0013 (16)	−0.0127 (13)
C4	0.0570 (19)	0.0442 (14)	0.080 (2)	−0.0043 (13)	−0.0023 (15)	−0.0085 (14)
C5	0.054 (2)	0.0367 (19)	0.111 (4)	0.000	0.005 (3)	0.000

### Geometric parameters (Å, º)

**Table:**

S1—C4	1.694 (3)	C2—H2	0.9300
S1—C1	1.701 (4)	C3—C4	1.363 (4)
O1—C5	1.225 (5)	C3—C5	1.464 (4)
C1—C2	1.347 (6)	C4—H4	0.9300
C1—H1	0.9300	C5—C3^i^	1.464 (4)
C2—C3	1.434 (5)		
			
C4—S1—C1	92.30 (18)	C4—C3—C5	126.5 (3)
C2—C1—S1	111.1 (3)	C2—C3—C5	123.1 (3)
C2—C1—H1	124.5	C3—C4—S1	112.6 (2)
S1—C1—H1	124.5	C3—C4—H4	123.7
C1—C2—C3	113.7 (3)	S1—C4—H4	123.7
C1—C2—H2	123.2	O1—C5—C3^i^	119.34 (17)
C3—C2—H2	123.2	O1—C5—C3	119.34 (17)
C4—C3—C2	110.3 (3)	C3^i^—C5—C3	121.3 (3)
			
C4—S1—C1—C2	−0.3 (3)	C1—S1—C4—C3	0.2 (3)
S1—C1—C2—C3	0.3 (4)	C4—C3—C5—O1	−155.2 (3)
C1—C2—C3—C4	−0.2 (4)	C2—C3—C5—O1	21.2 (3)
C1—C2—C3—C5	−177.1 (3)	C4—C3—C5—C3^i^	24.8 (3)
C2—C3—C4—S1	−0.1 (4)	C2—C3—C5—C3^i^	−158.8 (3)
C5—C3—C4—S1	176.8 (2)		

Symmetry code: (i) −*x*, *y*, −*z*+1/2.

### Hydrogen-bond geometry (Å, º)

**Table:**

*D*—H···*A*	*D*—H	H···*A*	*D*···*A*	*D*—H···*A*
C4—H4···O1^ii^	0.93	2.42	3.261 (4)	151

Symmetry code: (ii) *x*+1/2, *y*+1/2, −*z*+1/2.
